# Prediction of learning curves of wired and wireless intraoral scanners

**DOI:** 10.1038/s41598-023-48855-2

**Published:** 2023-12-08

**Authors:** Boncheol Koo, Keunbada Son, Ji-Min Lee, So-Yeun Kim, Myoung-Uk Jin, Kyu-Bok Lee

**Affiliations:** 1 Department of Prosthodontics, School of Dentistry, Kyungpook National University, 2177 Dalgubuldaero, Jung-gu, Daegu, 41940 Republic of Korea; 2 Advanced Dental Device Development Institute (A3DI), Kyungpook National University, Daegu, Republic of Korea; 3 Department of Dental Science, Graduate School, School of Dentistry, Kyungpook National University, Daegu, Republic of Korea; 4 Department of Conservative Dentistry, School of Dentistry, Kyungpook National University, Daegu, Republic of Korea

**Keywords:** Dentistry, Health care

## Abstract

This clinical study aimed to predict the learning curve of wireless and wired intraoral scanners (IOSs) and to compare the reduction patterns of working time. Overall, 14 participants were enrolled in the study. The intraoral scanning procedure was repeated four times, each using wireless and wired IOSs (i700; MEDIT). The work time from the first to the 600th iterations was predicted using the Wright model. Regarding statistical analysis, the Mann–Whitney U-test was performed for comparison between wireless and wired IOSs and between groups with and without an IOS usage experience, and the Friedman test was performed to evaluate the time reduction (α = 0.05). There was a significant difference between wireless and wired IOSs in the first (*P* = 0.008) and the third (*P* = 0.035) iterations. Moreover, the time for 600 iterations was statistically significantly different between wireless and wired IOSs (*P* < 0.05); however, there was no significant difference after the sixth iteration (e.g., seventh iteration: *P* = 0.062). In wireless IOS, no significant difference was found between participants with and without an IOS usage experience after the 34th iteration (*P* = 0.053). The difference in the learning effect between wireless and wired IOSs can be overcome by initial learning; however, an IOS usage experience can affect the learning time of wireless IOSs.

## Introduction

Recently, dental computer-aided design and computer-aided manufacturing (CAD/CAM) has been used in most dental clinics for fabricating dental prostheses^1–3^. Specifically, the chairside dental CAD/CAM process has been established for intraoral scanners (IOSs)^4–7^. Moreover, owing to the rapid development of IOSs, it has become possible to manufacture an accurate fixed dental prosthesis that that is at par with the prosthesis manufactured by conventional manufacturing methods^8–11^. For the optimal clinical application of IOSs in dentistry, the effects of temperature^9^, ambient light^10^, and narrow and deep area scans on the oral cavity^11^ were evaluated. Recently, the use of wireless IOSs has emerged as a method to address the limitations of movement by clinician owing to wires.

Learning of medical devices is conducted for stable use by inexperienced clinicians^12^. In order to evaluate the learning of dental CAD software, learning patterns were compared with learning curves for designing a dental prosthesis^13,14^. Additionally, the learning curves of different IOSs were compared to evaluate the effect of IOSs on working time^15,16^. Notably, the evaluation of the learning curve made it possible to recommend an appropriate amount of learning that enables the stable use of medical devices among clinicians without clinical experience^17–19^.

Learning curve refers to the tendency of a decrease in working time with an increase in the number of iterations for a specific task^20^. The learning curve has been found vary in the pattern of working time reduction depending on the type of medical device used and experience of the operator, even for the same task^21,22^. To evaluate the learning curve of a worker, many repeated learning events are required to be performed, but this process takes a lot of time of the worker; thus, deriving an accurate learning curve is difficult because of accumulated fatigue^23^. Therefore, the application of artificial intelligence algorithms or statistical methods for estimating time can be considered; however, this process also requires extensive data^24^. In the manufacturing industry, time reduction due to repeated learning among workers was derived using a mathematical formula^25–27^. The mathematical formula of the learning curve was first confirmed based on the phenomenon of production increase and cost decrease through repetitive work in the manufacturing time of an aircraft, and the model that formalized this was called the Wright model^28^. Notably, this model can estimate the long-term learning time of a worker, and it has been widely used owing to the accuracy of the results and a straightforward methodology^29–31^. In previous studies, the learning curve of dental CAD software was predicted using the Wright model, and differences in the learning effect between two software and among dental personnel were identified^13,14^.

Wireless IOSs were developed for the convenience of clinicians; however, their effect on learning has not been studied to date. This study aimed to perform four iterations of learning using wireless and wired IOSs and to compare the learning patterns by predicting a long-term learning curve using the Wright model. The null hypothesis of this study was that there was no difference in the learning time of wireless and wired IOSs, and the previous use of IOSs did not affect learning.

## Methods

This study was approved by the Institutional Review Board of Kyungpook National University Dental Hospital (approval number: KNUDH-2021-04-04-00, Date of approval: 28/05/2021) and registered in the Institutional Review Board of Kyungpook National University Dental Hospital before patient recruitment. Overall, 14 participants were enrolled for the comparison of wireless and wired IOSs. All patients provided written informed consent. This study was conducted in accordance with the Helsinki Declaration and the Good Clinical Practice guidelines and reported the findings based on the applicable Consolidated Standard of Reporting Trials guidelines.

The total number of participants was calculated using power analysis software (G*Power version 3.1.9.2; Heinrich-Heine-Universität Düsseldorf, Düsseldorf, Germany) based on the results of three pilot experiments [effect size (d) = 1.5475; actual power = 99%; power = 99%; α = 0.05]. In total, 14 right-handed dentists, including 8 men and 6 women, who had no previous history of musculoskeletal disorders were enrolled in this study. The mean age and dental clinical experience of the participants was 29.7 ± 4.1 years and 3.0 ± 1.5 years, respectively. Among the participants, seven were familiar with the usage of IOSs and were thus allocated to the group with IOSs experience. The remaining seven participants, having no prior IOSs experience, were assigned to the other group. Notably, the group with experience was not limited to using the specific IOSs used in this study but had diverse experiences with various IOSs, including branded products.

Wireless and wired IOSs (i700; MEDIT, Seoul, Republic of Korea) were used for the analysis. According to the manufacturer, wireless IOSs have an added module for wireless data transmission to the same optical system as wired IOSs. Given that the computer specifications can greatly affect the scanning speed, a computer system with specifications better than the manufacturer’s recommended specifications was used in our study (Table 1). All experiments of wireless and wired IOSs (i700; MEDIT, Seoul, Republic of Korea) were performed using the same computer with the same software version (MEDIT, Seoul, Republic of Korea). The wireless IOS system comprises a transmitter integrated into the scanner and a receiver within the wireless hub. Data and control commands are transmitted using two distinct frequencies: 60 GHz for data transmission and 2.4 GHz for scanner control. The manufacturer asserts that the data transfer speed remains constant and is not affected by prolonged working times or continuous data accumulation during intraoral scanning. This consistency in transfer speed is attributed to the rendering of scanned data by the software installed on the laptop. Since both wireless and wired IOSs utilize the same laptop and software for data rendering, it is posited that the wireless IOS's data transfer speeds remain unaffected even over extended periods of use.

**Table 1:** Specification of the computer system used.

System	Specification
Operating System	Windows 10 Pro 64-bit
Processor	AMD Ryzen 9 5900HX with Radeon Graphics (16 CPUs), ~ 3.3 GHz
Memory	32768 MB RAM
Graphics Card	NVIDIA GeForce RTX 3070 Laptop GPU (Memory: 24,123 MB)

A dental mannequin (Simple Manikin III; NISSIN, Kyoto, Japan) including artificial head with rubber sheets for cheeks and a maxillary and mandibular typodont model (D85DP-500B.1; NISSIN) were installed in the dental unit chair system (N2; MEGAGEN, Daegu, Republic of Korea), and each participant took part in the intraoral scan process. Further, the patient’s chair and dental stool were adjusted to avoid discomfort.

A total of 14 participants received hands-on training on how to operate the two types of IOSs and practiced once per IOS for approximately 30 min. The experiment order was randomly assigned to each participant, and a break of > 3 h was taken between the use of each IOS. Four iterations were performed for wireless and wired IOSs per participant. To prevent the accumulation of muscle fatigue in each iteration, a break of ≥ 10 min was taken before moving on to the next round, and when the participant did not feel fatigued, the next round of work was performed. During the scanning process, the mandible was scanned after the maxillary of the typodont model was scanned. As for the scanning strategy, the maxillary complete arch was scanned in the occlusal and incisal directions from the maxillary left second molar to the right second molar and was successively scanned in the buccal direction and finished in the lingual direction. Subsequently, the mandibular complete arch performed the same scanning strategy as the maxillary. Finally, the bite scan process was performed. During the scanning process, the participants continuously checked for defects in the scanned area and completed the scan of the complete arch so that there were no holes in the tooth area. All scan times were recorded in seconds by one investigator. Another investigator checked the scanning process of the participants and monitored the use of inappropriate IOSs, and determined that the scan was completed. After all the experiments, the participants were assessed about the type of IOSs they preferred and their reason.

For the learning time of the fourth iteration, the learning time up to the 600th iteration was predicted using the Wright model. The Wright model used the following formula^13,14,29^:

$${Y}_{\left(x\right)}={Y}_{1}{X}^{b}$$

It represents the working time in the Xth iteration, Y_1_ represents the first working time, *b* represents the slope of each worker’s learning curve, and the following formula was used:

$$b={\text{log}}\, learning \, rate/\mathrm{log }2$$

The learning rate indicates the degree of improvement in the work time of the next iteration compared with the work time of the previous iteration. *b* is a logarithmic function, which indicates the rate of learning and rapid adaptation to task performance^29^.

All data were analyzed using statistics software (IBM SPSS ver 23.0; IBM Corp, Chicago, IL, USA). Data distribution was verified using the Shapiro–Wilk test, and we found that the data did not conform to a normal distribution. Therefore, the Mann–Whitney U-test was performed to compare wireless and wired IOSs and groups with and without an IOS usage experience. The Friedman test was performed to evaluate the time reduction of each IOS (α = 0.05). For the post hoc test of the fourth iteration, the Mann–Whitney U-test and Bonferroni correction method were used (α = 0.0083).

## Results

**Figure 1:**
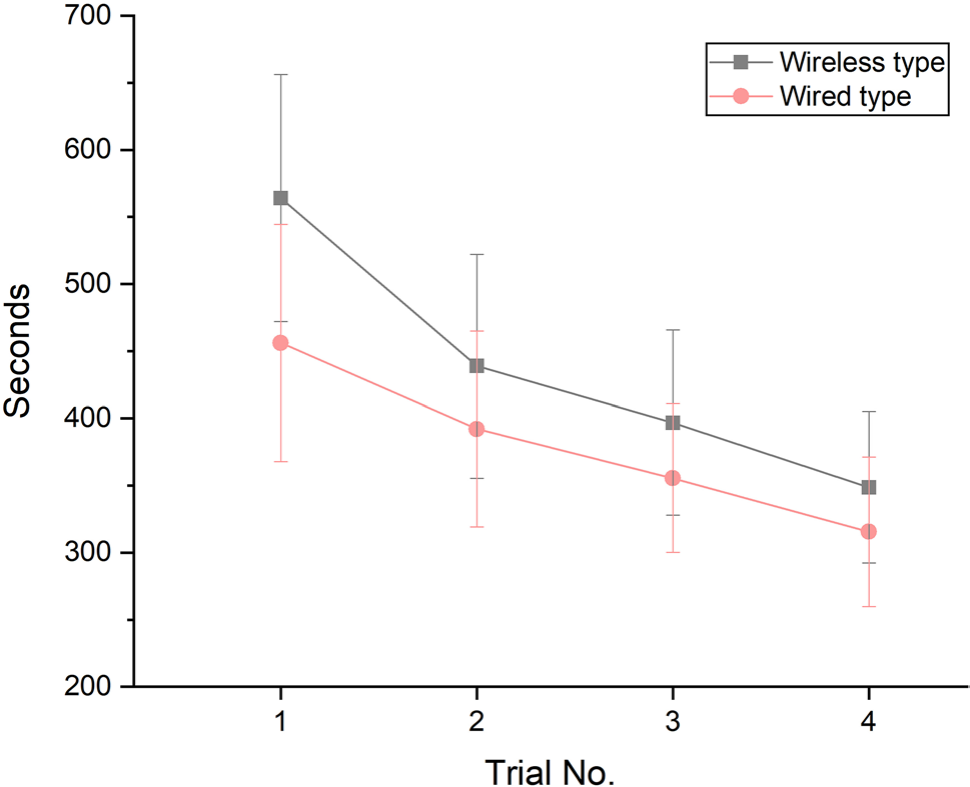
Learning curve showing the mean working times for wireless and wired IOSs in the first, second, third, and fourth iterations.

**Figure 2:**
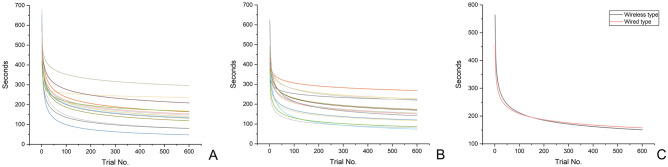
Prediction of learning curves of the working time of wireless and wired IOSs using the learning curve model. (**A**) Wireless IOS, (**B**) wired IOS, (**C**) mean learning curves of wireless and wired IOSs.

Both wireless and wired IOSs were confirmed to significantly reduce time by repeated learning (Table 2, Fig. 1) (*P* < 0.001). Across the span of four repetitive learning iterations, notable differences in the learning times between the two types of IOSs were observed in the first trial (*P* = 0.008) and the third trial (*P* = 0.035) (Table 2). In the predicted 600th learning curve, a significant difference in the two IOSs was found from the first to the sixth iterative learning (Table 3, Fig. 2) (*P* < 0.05); however, no significant difference was found between the wireless and wired IOSs from the seventh iterative learning (Table 3, Fig. 2) (*P* = 0.062).

**Table 2:** Comparison of working time (seconds) for wireless and wired IOSs in the first, second, third, and fourth iterations (N = 14 per IOS type).

Trial no	IOS type	Mean	SD	Median	95% confidence interval		*P*	*P*			
					Lower limit	Upper limit		Wireless IOS		Wired IOS	
1	Wireless IOS	564.07	92.13	580.50	515.80	612.33	0.008*	< 0.001 **	A	< 0.001 **	A
	Wired IOS	456.14	88.41	462.50	409.82	502.45					
2	Wireless IOS	438.78	83.37	462.00	395.11	482.45	0.077		AB		AB
	Wired IOS	392.07	72.79	415.00	353.94	430.20					
3	Wireless IOS	396.85	69.02	413.00	360.70	433.01	0.035*		B		BC
	Wired IOS	355.50	55.37	373.00	326.49	384.50					
4	Wireless IOS	348.71	56.42	358.00	319.15	378.27	0.194		B		C
	Wired IOS	315.57	55.73	326.50	286.37	344.76					

*IOS* intraoral scanner.

*Significant difference between two IOSs determined by the Mann–Whitney U-test, *P* < 0.05.

**Significant difference determined by the Friedman test, *P* < 0.05.

Same capital letters (column) are not significantly different in fourth iterations according to the Mann–Whitney U-test and the Bonferroni correction method (*P* < 0.0083).

**Table 3:** Comparison of the working times (seconds) for wireless and wired intraoral scanners using the learning curve model (N = 14 per IOS type).

Trial no	Wireless IOS					Wired IOS					*P*
	Mean	SD	Median	95% confidence interval		Mean	SD	Median	95% confidence interval		
				Lower limit	Upper limit				Lower limit	Upper limit	
1	564.07	92.13	580.50	515.80	612.33	456.14	88.41	462.50	409.82	502.45	0.008*
2	482.08	64.43	501.05	448.33	515.84	403.07	69.48	416.92	366.67	439.46	0.004*
3	440.48	55.42	456.53	411.45	469.52	375.32	62.58	395.98	342.54	408.10	0.006*
4	413.45	51.85	426.60	386.29	440.62	356.97	59.27	378.18	325.92	388.02	0.012*
5	393.80	50.43	404.12	367.38	420.22	343.44	57.47	363.13	313.33	373.55	0.019*
6	378.53	49.96	388.12	352.36	404.71	332.82	56.42	346.62	303.26	362.38	0.031*
7	366.16	49.96	374.65	339.99	392.33	324.14	55.80	334.45	294.91	353.37	0.062
8	355.81	50.19	363.21	329.52	382.11	316.83	55.42	326.68	287.80	345.86	0.114
9	346.97	50.54	353.42	320.49	373.44	310.54	55.20	319.98	281.62	339.46	0.150
10	339.27	50.94	344.88	312.59	365.96	305.03	55.07	314.11	276.18	333.89	0.194
591	150.96	64.71	144.78	117.07	184.86	158.09	60.04	153.25	126.64	189.54	0.667
592	150.92	64.71	144.73	117.02	184.82	158.05	60.04	153.20	126.60	189.50	0.667
593	150.87	64.71	144.67	116.97	184.77	158.01	60.04	153.15	126.56	189.46	0.667
594	150.82	64.71	144.62	116.93	184.72	157.97	60.04	153.11	126.52	189.42	0.667
595	150.78	64.71	144.57	116.88	184.67	157.93	60.04	153.06	126.48	189.38	0.667
596	150.73	64.71	144.51	116.83	184.63	157.89	60.04	153.02	126.43	189.34	0.667
597	150.68	64.71	144.46	116.79	184.58	157.85	60.04	152.97	126.39	189.30	0.667
598	150.64	64.71	144.41	116.74	184.53	157.81	60.04	152.93	126.35	189.26	0.667
599	150.59	64.71	144.35	116.69	184.49	157.76	60.04	152.88	126.31	189.22	0.667
600	150.54	64.71	144.30	116.65	184.44	157.72	60.04	152.84	126.27	189.18	0.667
*P*	< 0.001**					< 0.001**					

*Significant difference between two IOSs determined by the Mann–Whitney U-test, *P* < 0.05.

**Significant difference determined by the Friedman test, *P* < 0.05.

**Figure 3:**
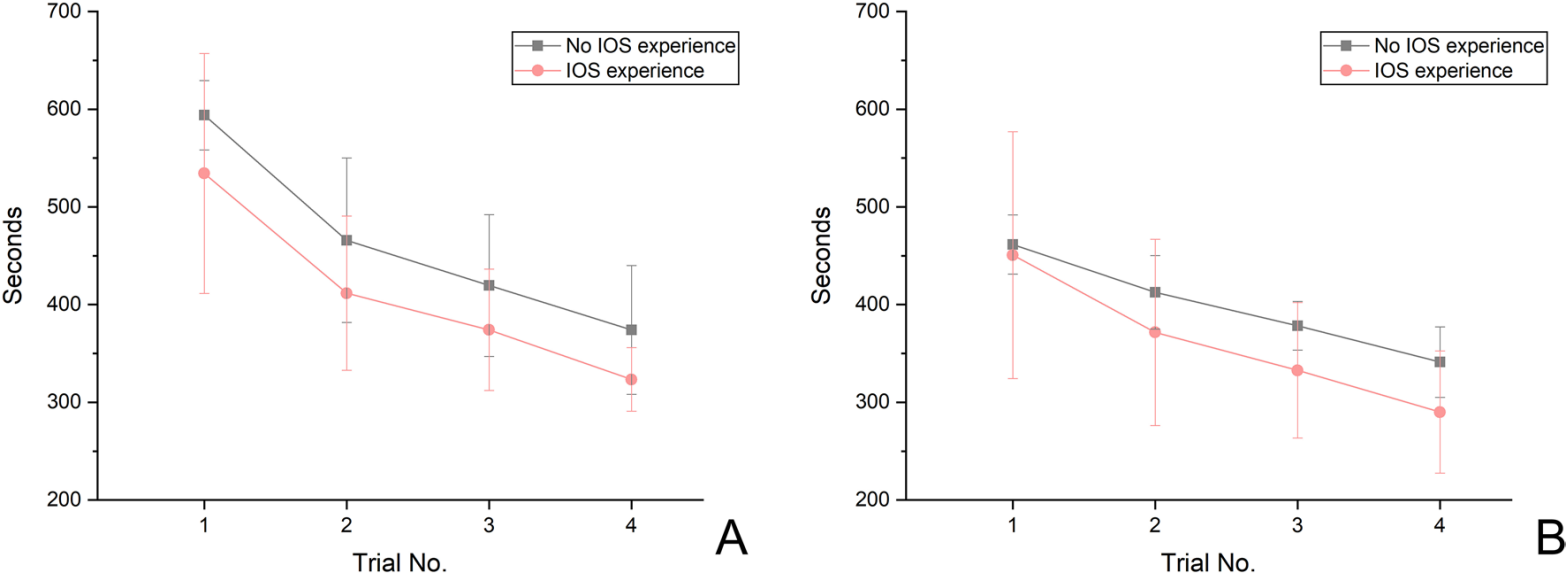
Learning curves showing the mean working times of wireless and wired intraoral scanners in the first, second, third, and fourth iterations in terms of presence or absence of IOS usage experience. (**A**) Wireless IOS, (**B**) wired IOS.

**Figure 4:**
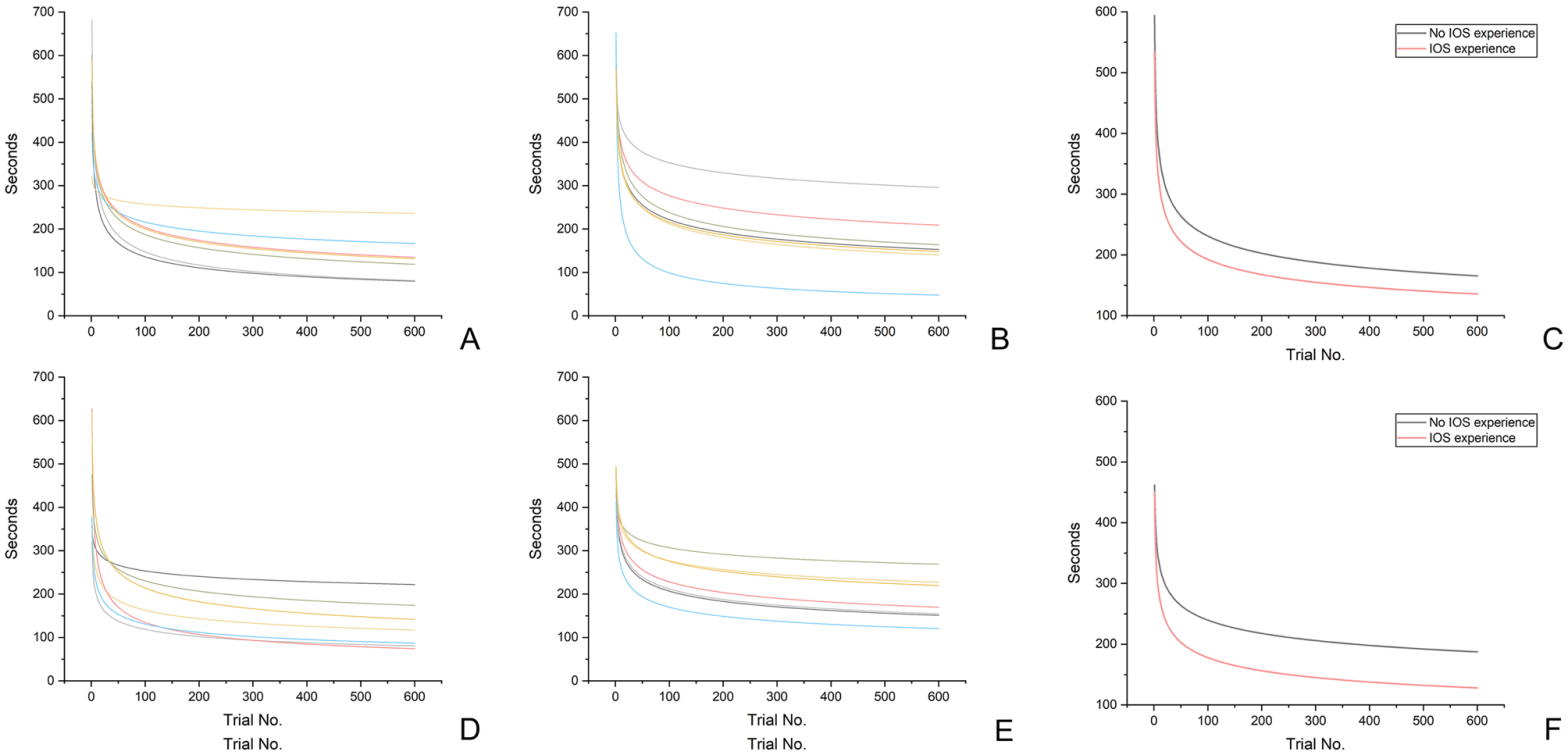
Prediction of the learning curves of the working times of wireless and wired IOSs in the first, second, third, and fourth iterations in terms of the presence or absence of IOS usage experience. (**A**) Without IOS usage experience (wireless IOS), (**B**) with IOS usage experience (wireless IOS), (**C**) mean learning curve (wireless IOS), (**D**) without IOS usage experience (wired IOS), (**E**) with IOS usage experience (wired IOS), (**F**) mean learning curve (wired IOS).

The group with an IOS usage experience demonstrated a significantly lower working time than the group without experience only in the fourth iteration learning of wireless IOSs (Table 4, Fig. 3) (*P* = 0.026). Moreover, regardless of the IOS usage experience, all participants confirmed that there was a significant decrease in working time (Table 4, Fig. 3) (*P* < 0.05). In the learning curve of 600 iterations predicted from wireless IOSs, in the repeated learning from the 1th to 34th iteration, the group with an IOS usage experience demonstrated a significantly lower learning time than the group without an IOS usage experience (Table 5, Fig. 4) (*P* < 0.05); in addition, the significant difference in learning time disappeared from the 35th iteration (Table 5, Fig. 4) (*P* = 0.053). However, in the 600th learning curve predicted by wired IOSs, no significant difference was found in learning time because of the presence or absence of an IOS usage experience (Table 6, Fig. 4) (*P* > 0.05).

**Table 4:** Comparison of the mean working time (seconds) for wireless and wired intraoral scanners in the first, second, third, and fourth iterations between participants with and without an intraoral scanner usage experience (N = 7 per usage experience group).

Trial no	IOS usage experience	IOS type	Mean	SD	Median	95% confidence interval		*P*		*P*			
						Lower limit	Upper limit	Wireless	Wired	Wireless IOS		Wired IOS	
1	No IOS experience group	Wireless IOS	593.86	35.61	574.00	575.21	612.51	0.535	0.456	No IOS experience group < 0.001**	AB	No IOS experience group 0.008**	A
		Wired IOS	461.57	30.19	464.00	445.76	477.38						
	IOS experience group	Wireless IOS	534.29	122.70	587.00	470.01	598.56				BC		AB
		Wired IOS	450.71	126.33	382.00	384.54	516.89						
2	No IOS experience group	Wireless IOS	465.86	84.25	480.00	421.73	509.99	0.259	0.535		C		AB
		Wired IOS	412.57	37.73	416.00	392.81	432.33						
	IOS experience group	Wireless IOS	411.71	79.07	422.00	370.30	453.13				C		B
		Wired IOS	371.57	95.27	327.00	321.66	421.48						
3	No IOS experience group	Wireless IOS	419.57	72.50	431.00	381.59	457.55	.097	.259	IOS experience group < 0.001**	AB	IOS experience group 0.037**	A
		Wired IOS	378.29	24.86	392.00	365.26	391.31						
	IOS experience group	Wireless IOS	374.14	62.15	400.00	341.59	406.70				BC		AB
		Wired IOS	332.71	69.38	321.00	296.37	369.06						
4	No IOS experience group	Wireless IOS	374.00	65.93	378.00	339.46	408.54	0.026*	0.128		CD		AB
		Wired IOS	341.14	36.10	347.00	322.23	360.05						
	IOS experience group	Wireless IOS	323.43	32.55	310.00	306.38	340.48				D		B
		Wired IOS	290.00	62.47	305.00	257.28	322.72						

*IOS* intraoral scanner.

*Significant difference between the groups with and without IOS usage experience determined by the Mann–Whitney U-test, *P* < 0.05.

**Significant difference determined by the Friedman test, *P* < 0.05.

Same capital letters (column) are not significantly different in the fourth iterations according to the Mann–Whitney U-test and the Bonferroni correction method (*P* < 0.0083).

**Table 5:** Comparison of the working times (seconds) for wireless intraoral scanners using the learning curve model between participants with and without an intraoral scanner usage experience (N = 7 per usage experience group).

Trial no	No IOS experience group					IOS experience group					*P*
	Mean	SD	Median	95% confidence interval		Mean	SD	Median	95% confidence interval		
				Lower limit	Upper limit				Lower limit	Upper limit	
1	593.86	35.61	574.00	575.21	612.51	534.29	122.70	587.00	470.01	598.56	0.535
2	510.36	18.22	513.66	500.82	519.91	453.82	82.46	499.31	410.62	497.01	0.318
3	467.88	28.94	476.55	452.72	483.04	413.09	63.78	454.21	379.68	446.50	0.073
4	440.22	37.29	445.95	420.69	459.76	386.69	52.58	423.58	359.15	414.24	0.026*
5	420.07	43.22	423.58	397.44	442.71	367.53	45.08	398.84	343.92	391.15	0.011*
6	404.40	47.61	406.13	379.46	429.35	352.68	39.77	375.38	331.84	373.51	0.026*
7	391.68	51.02	391.94	364.96	418.40	340.65	35.88	356.63	321.85	359.44	0.017*
8	381.03	53.73	380.06	352.89	409.18	330.61	32.99	341.15	313.32	347.89	0.026*
9	371.92	55.96	369.87	342.61	401.23	322.03	30.84	328.05	305.87	338.18	0.026*
10	363.98	57.83	360.99	333.69	394.27	314.57	29.24	316.76	299.25	329.89	0.026*
11	356.97	59.41	353.14	325.85	388.09	308.00	28.07	306.88	293.29	322.70	0.026*
12	350.70	60.77	346.12	318.86	382.53	302.13	27.23	298.13	287.87	316.40	0.026*
13	345.05	61.96	339.79	312.59	377.50	296.85	26.65	290.34	282.90	310.81	0.026*
14	339.91	63.01	334.03	306.90	372.91	292.06	26.27	287.24	278.30	305.82	0.026*
15	335.20	63.94	328.75	301.71	368.69	287.68	26.05	284.38	274.03	301.32	0.026*
16	330.87	64.77	323.90	296.94	364.79	283.65	25.95	281.74	270.05	297.24	0.026*
17	326.85	65.51	319.65	292.54	361.17	279.92	25.95	280.25	266.33	293.51	0.026*
18	323.12	66.19	315.89	288.45	357.79	276.46	26.03	279.48	262.82	290.09	0.026*
19	319.64	66.80	312.38	284.65	354.63	273.23	26.16	278.76	259.52	286.93	0.026*
20	316.37	67.36	309.09	281.09	351.66	270.21	26.34	278.07	256.41	284.01	0.026*
21	313.30	67.87	305.99	277.75	348.85	267.37	26.56	277.42	253.45	281.28	0.026*
22	310.41	68.34	303.06	274.61	346.20	264.69	26.80	274.92	250.65	278.73	0.026*
23	307.67	68.78	300.29	271.64	343.69	262.17	27.06	271.83	247.99	276.34	0.026*
24	305.07	69.18	297.66	268.83	341.31	259.77	27.34	268.92	245.45	274.09	0.026*
25	302.61	69.55	295.16	266.17	339.04	257.50	27.62	266.15	243.03	271.97	0.026*
26	300.26	69.90	292.78	263.64	336.87	255.34	27.91	263.51	240.72	269.96	0.026*
27	298.02	70.22	290.51	261.23	334.80	253.28	28.21	261.17	238.51	268.06	0.026*
28	295.88	70.53	288.33	258.94	332.82	251.32	28.51	259.80	236.38	266.25	0.026*
29	293.83	70.81	286.25	256.74	330.92	249.44	28.81	258.48	234.35	264.53	0.026*
30	291.87	71.08	284.25	254.64	329.10	247.64	29.10	257.22	232.39	262.88	0.026*
31	289.98	71.33	282.33	252.62	327.35	245.91	29.40	256.00	230.51	261.31	0.026*
32	288.17	71.56	280.48	250.69	325.66	244.25	29.69	254.82	228.70	259.81	0.026*
33	286.43	71.79	278.71	248.83	324.03	242.66	29.98	253.69	226.95	258.37	0.026*
34	284.75	72.00	276.99	247.04	322.47	241.12	30.27	252.59	225.27	256.98	0.038*
35	283.13	72.20	275.34	245.31	320.95	239.64	30.55	251.53	223.64	255.65	0.053
36	281.57	72.38	273.74	243.65	319.48	238.22	30.83	250.51	222.07	254.36	0.053
37	280.06	72.56	272.19	242.05	318.07	236.84	31.10	249.52	220.55	253.13	0.053
38	278.59	72.73	270.70	240.50	316.69	235.50	31.37	248.56	219.07	251.93	0.053
39	277.18	72.89	269.25	239.00	315.36	234.21	31.63	247.62	217.65	250.78	0.073
40	275.81	73.05	267.85	237.54	314.07	232.96	31.89	246.72	216.26	249.67	0.073
591	165.91	75.17	153.54	126.53	205.28	136.03	53.87	132.33	107.81	164.25	0.318
592	165.86	75.16	153.49	126.48	205.23	135.99	53.88	132.27	107.76	164.21	0.318
593	165.81	75.16	153.44	126.43	205.18	135.94	53.89	132.22	107.72	164.17	0.318
594	165.75	75.16	153.38	126.39	205.12	135.90	53.90	132.17	107.67	164.14	0.318
595	165.70	75.15	153.33	126.34	205.07	135.86	53.91	132.12	107.62	164.10	0.318
596	165.65	75.15	153.28	126.29	205.02	135.82	53.91	132.07	107.57	164.06	0.318
597	165.60	75.14	153.22	126.24	204.96	135.77	53.92	132.02	107.53	164.02	0.318
598	165.55	75.14	153.17	126.19	204.91	135.73	53.93	131.96	107.48	163.98	0.318
599	165.50	75.14	153.12	126.14	204.86	135.69	53.94	131.91	107.43	163.94	0.318
600	165.45	75.13	153.06	126.10	204.81	135.65	53.95	131.86	107.39	163.90	0.318
*P*	< 0.001**					< 0.001**					

*IOS* intraoral scanner.

*Significant difference between the groups with and without an IOS usage experience determined by the Mann–Whitney U-test, *P* < 0.05.

**Significant difference determined by the Friedman test, *P* < 0.05.

**Table 6:** Comparison of working times (seconds) of wired intraoral scanners using the learning curve model between participants with and without an intraoral scanner usage experience (N = 7 per usage experience group).

Trial no	No IOS experience group					IOS experience group					*P*
	Mean	SD	Median	95% confidence interval		Mean	SD	Median	95% confidence interval		
				Lower limit	Upper limit				Lower limit	Upper limit	
1	461.57	30.19	464.00	445.76	477.38	450.71	126.33	382.00	384.54	516.89	0456
2	417.30	29.01	422.97	402.11	432.50	388.84	95.63	338.24	338.75	438.93	0.456
3	393.54	30.26	398.20	377.69	409.39	357.12	82.44	328.27	313.93	400.30	0.456
4	377.56	31.65	389.43	360.98	394.14	336.39	74.98	321.37	297.11	375.66	0.318
5	365.65	32.90	375.29	348.42	382.88	321.24	70.18	316.12	284.48	358.00	0.209
6	356.21	33.98	364.12	338.41	374.01	309.44	66.85	311.89	274.43	344.46	0.165
7	348.44	34.92	354.93	330.15	366.73	299.85	64.42	308.36	266.10	333.60	0.128
8	341.86	35.75	347.17	323.13	360.58	291.81	62.59	305.34	259.03	324.60	0.128
9	336.16	36.47	340.46	317.06	355.27	284.93	61.16	301.18	252.89	316.97	0.097
10	331.15	37.12	334.56	311.71	350.60	278.92	60.03	290.77	247.48	310.37	0.073
591	187.78	52.34	170.18	160.36	215.19	128.41	54.92	117.39	99.65	157.18	0.073
592	187.73	52.34	170.13	160.31	215.15	128.38	54.92	117.35	99.61	157.14	0.073
593	187.69	52.35	170.08	160.27	215.11	128.34	54.92	117.31	99.57	157.11	0.073
594	187.65	52.35	170.03	160.23	215.07	128.30	54.92	117.28	99.53	157.07	0.073
595	187.60	52.35	169.99	160.18	215.03	128.26	54.92	117.24	99.49	157.03	0.073
596	187.56	52.35	169.94	160.14	214.98	128.22	54.92	117.20	99.45	156.99	0.073
597	187.52	52.35	169.89	160.09	214.94	128.18	54.92	117.17	99.41	156.95	0.073
598	187.48	52.36	169.84	160.05	214.90	128.14	54.92	117.13	99.38	156.91	0.073
599	187.43	52.36	169.80	160.01	214.86	128.11	54.92	117.10	99.34	156.87	0.073
600	187.39	52.36	169.75	159.96	214.82	128.07	54.92	117.06	99.30	156.84	0.073
*P*	< 0.001**					< 0.001**					

*IOS* intraoral scanner.

*Significant difference between the groups with and without an IOS usage experience determined by the Mann–Whitney U-test, *P* < 0.05.

**Significant difference determined by the Friedman test, *P* < 0.05.

**Figure 5:**
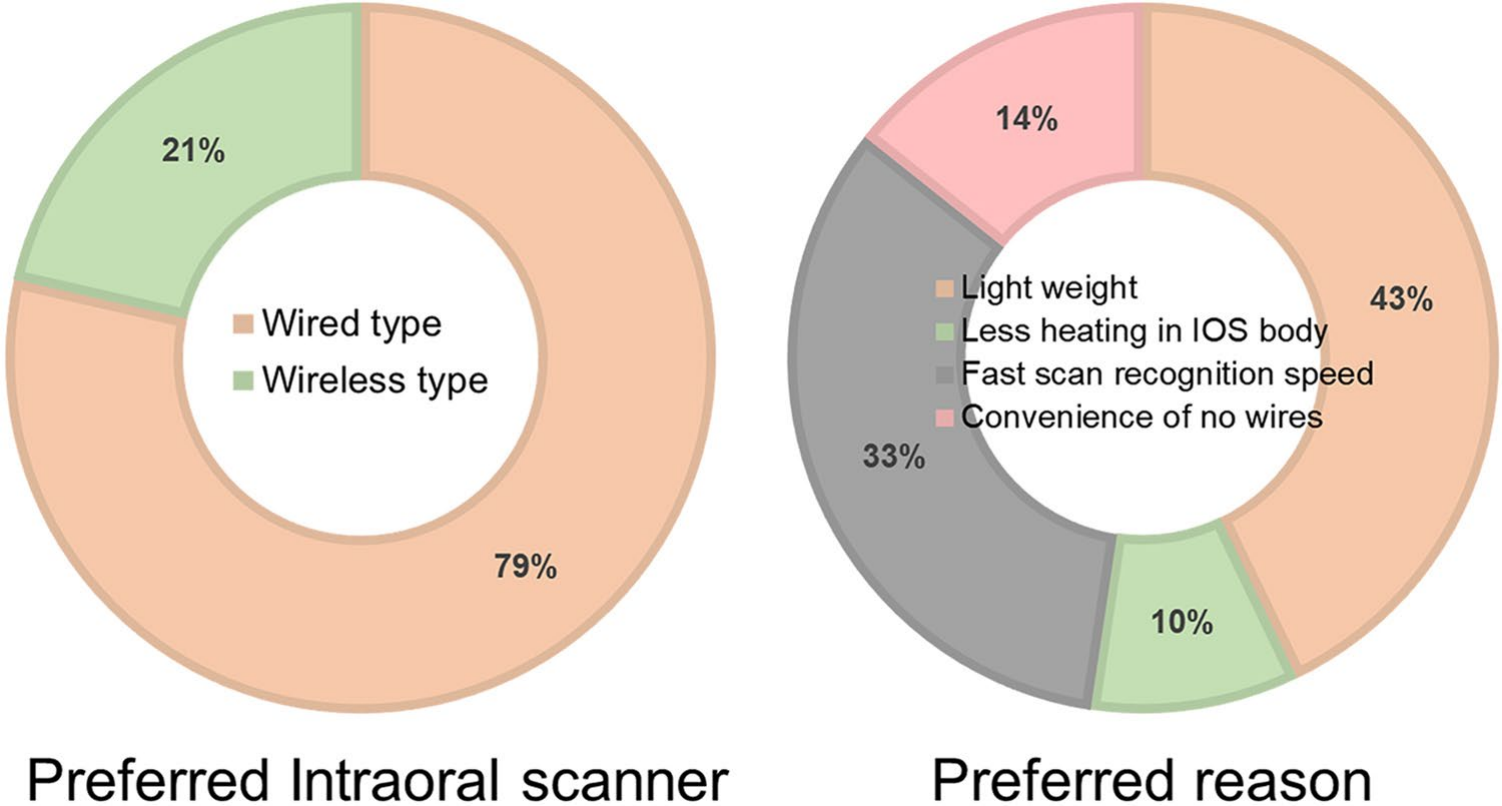
Preference and reasons for the use of wireless and wired intraoral scanners.

Moreover, 79% of the 14 participants preferred wired IOSs (Fig. 5) because of a lighter weight (43%), fast scan recognition speed (33%), and less heating on the IOS body (10%) than the wireless type. Wireless IOSs were preferred by 21% of the participants (Fig. 5) because of convenience without wires (14%).

## Discussion

The present study aimed to predict the learning curves of wireless and wired IOSs and to compare the time reduction patterns of operators in repetitive learning. The learning curves of wireless and wired IOSs revealed a significant decrease in time in the early stage of learning, and after the sixth repeated learning, the learning curves revealed no significant difference (Table 3, Fig. 2) (*P* = 0.062). Moreover, the learning curve was compared according to the participant’s experience of using IOS. Those who had no IOS usage experience had difficulty learning wireless IOSs, and during long-term experiments (34th repeated learning), a significant difference was found between the learning curves of such participants and participants who had IOS usage experience (Table 5, Fig. 4) (*P* < 0.05). However, in wired IOSs, no difference in the learning curve was found because of IOS usage experience (Table 6, Fig. 4) (*P* > 0.05). Therefore, the null hypothesis was partially rejected (*P* < 0.05).

In the manufacturing industry, the learning curve was evaluated for the worker’s efficiency of working, and in the medical field, for determining the proficiency of new technology or medical device, the time reduction pattern was analyzed^12,18^. A previous study evaluated the learning curve of an IOS and found that dental clinical experience affected the learning effect^16^. Similarly, in the present study, a significant time reduction was confirmed by repetitive learning for both wireless and wired IOSs (Table 2) (*P* < 0.001), and the learning effect differed according to the use of the wireless or wired IOS (*P* < 0.05).

In a previous study, the learning curve was predicted by repeatedly learning the design process of a dental prosthesis using dental CAD software^13,14^. No difference was found in working time between the two types of software through long-term repetitive learning (> 50 times)^13^. In the present study, unlike the previous study^13^, both IOSs were obtained from the same manufacturer, and they were composed of the same hardware, with the difference only in terms of being wired and wireless. Therefore, in this study, the difference between wired and wireless IOSs did not significantly impact the learning effect. In contrast, another previous study reported that the learning of dental CAD software had the same effect depending on the dental personnel after the initial repetitive learning^14^. Moreover, in our study, learning with wireless IOS was difficult in the absence of IOS usage experience. After long-term experiments (34th repeated learning), the participants with no IOS usage experience demonstrated learning effects similar to those with usage experience (Table 3). This result can be attributed to the difference in difficulty level between the CAD design of dental prosthesis and the intraoral scan. Dental CAD software is composed of simple work sequences to ensure that first-time users can easily design the prosthesis; however, regarding the process of intraoral scanning, it is initially difficult for users to insert and move the IOS probe into the oral cavity and continuously check the virtual cast that is transmitted to the computer and displayed on the software during the process. In the present study, in wired IOSs, factors of IOS experience did not impact the learning effect, and no differences were noted in the learning curves (Table 6, Fig. 4) (*P* > 0.05). Therefore, learning of wireless IOSs was more difficult than that of wired IOS for first-time users. The manufacturer of wireless IOSs asserts that, despite prolonged intraoral scanning periods and continuous data accumulation, the data transfer speed remains constant. However, the operator must simultaneously monitor the virtual cast formation on the laptop during the scanning process. Interestingly, our survey results showed a preference for wired IOSs due to their faster scan recognition speed, suggesting a perceptible lag in scan recognition with wireless IOSs. This implies that although the wireless IOS maintains consistent data transfer speed, the immediate rendering and presentation on the laptop may be slower. Consequently, this could be seen as a drawback for wireless IOSs, particularly since dentists need to quickly assess scan progress on the laptop monitor during procedures.

A previous study reported that repeated use of IOSs could affect muscle fatigue^8^. This is one of the heaviest devices among medical devices used directly in the oral cavity, and the weight of IOSs ranged from 113 to 585 g^8^. In the present study, a survey was conducted after the experiment, and 79% of the participants preferred wired IOSs (Fig. 5). Participants cited lighter weight (43%), faster scan recognition speed (33%), and less heating on the IOS body (10%), which are the reasons that learning of wireless IOSs was more difficult than that of wired IOSs for first-time users. Participants had difficulties using wireless IOSs because of heaviness, and the scan recognition speed was affected by data transmission delay in wireless data transmission. The weight of wireless IOSs increased by 25.3% compared with the wired type because of the module for wireless transmission of data and battery (wired IOS, 245 g; wireless IOS, 328 g). This feature of wireless IOSs needs to be improved to provide an optimal learning effect on first-time users. Conversely, 21% of participants who favored wireless IOSs highlighted the convenience of a cordless design as a primary advantage. The varied intraoral cavity access environments encountered by dentists necessitate adaptability in positioning, an area where wireless IOSs can potentially enhance a worker's mobility and ease of operation.

This study has minor limitations. An existing learning model was applied in the manufacturing industry to evaluate the learning effect; however, the physical condition of the worker on the day of the experiment was not considered. To improve the accuracy of the predicted learning curve, further studies are needed to improve the formula through long-term evaluation. In addition, a wireless IOS from one manufacturer was used. Further studies evaluating various IOSs are needed. However, not many manufacturers release and sell wireless IOSs, and if they can be improved based on the results of this study, it is expected to provide optimal learning effects on first-time users.

## Conclusion

This clinical study has demonstrated that a wired IOS requires less time than a wireless IOS at the beginning of use. However, the reduction patterns were found to be similar after short-term experiments (sixth repeated learning). Participants without prior IOS usage experience encountered difficulties in learning to use a wireless IOS. However, after long-term experiments (34th repeated learning), their learning time was observed to be the same as those with prior IOS experience. Thus, it can be concluded that wireless IOSs require more learning time for first-time users compared to wired IOSs. This difference in learning curves can be attributed to the weight of wireless IOSs and the scanning delay due to wireless data transmission. To provide optimal learning effects for first-time users, it is recommended that improvements be made to wireless IOSs. Currently, various wireless IOSs are being developed, and further long-term clinical studies using wireless IOSs from different manufacturers are required.

## Data Availability

All outcome data are available as summary measures or representative images in the main text or the extended data. The raw datasets generated analyzed during the current study are available from the corresponding author on reasonable request.
